# Takayasu Arteritis

**DOI:** 10.3389/fped.2018.00265

**Published:** 2018-09-24

**Authors:** Ricardo A. G. Russo, María M. Katsicas

**Affiliations:** Service of Immunology & Rheumatology, Hospital de Pediatría “Prof. Dr. Juan P. Garrahan”, Buenos Aires, Argentina

**Keywords:** Takayasu, arteritis, vasculitis, aorta, large vessel, children

## Abstract

Takayasu arteritis is an idiopathic granulomatous vasculitis of the aorta and its main branches and it constitutes one of the more common vasculitides in children. Inflammation and intimal proliferation lead to wall thickening, stenotic or occlusive lesions, and thrombosis, while destruction of the elastica and muscularis layers originates aneurysms and dissection. Carotid artery tenderness, claudication, ocular disturbances, central nervous system abnormalities, and weakening of pulses are the most frequent clinical features. The diagnosis is usually confirmed by the observation of large vessel wall abnormalities: stenosis, aneurysms, occlusion, and evidence of increased collateral circulation in angiography, MRA or CTA imaging. The purpose of this revision is to address the current knowledge on pathogenesis, investigations, classification, outcome measures and management, and to emphasize the need for timely diagnosis, effective therapeutic intervention, and close monitoring of this severe condition.

## Introduction

Takayasu arteritis (TA) is an idiopathic, granulomatous, large-vessel arteritis that predominantly involves the aorta, its major branch arteries, and (less frequently) the pulmonary arteries (1). The disease has been referred to with a number of different names in the past, such as aortic arch syndrome, pulseless disease, idiopathic aortitis, stenosing aortitis, aortoarteritis, and occlusive thromboarteriopathy. The Chapel Hill Consensus Conference defined TA as “a granulomatous inflammation of the aorta and its branches ‘usually’ occurring in patients younger than 50 years” (2). The disease is named after Mikito Takayasu, a Japanese ophthalmologist, who first described the arterio-venous anomalies in the retina of a patient with the disease in 1908. Inflammation and endothelial damage usually lead to wall thickening, thrombus formation, stenotic and occlusive lesions, while destruction of the muscularis and elastic layers originates dilatation and aneurysms. These lesions often result in organ dysfunction secondary to ischemia (3). It is the most common cause of granulomatous inflammation of large arteries and the third most common cause of vasculitis in the pediatric age group (4). Paucity of specific symptoms and laboratory biomarkers, as well as difficulties in assessing disease activity and progression, make the disease often unrecognized at onset, and its activity frequently underestimated. The disease course is commonly persistently active, allowing silent damage accrual and significant short- and long-term morbidity and mortality. However, early diagnosis and integrative management principles have led to better survival rates.

## Epidemiology

TA has been recognized worldwide. Its overall incidence has been estimated to be 2/1,000,000 per year (1, 5). It is more prevalent in Central and South America, Africa, India, and the Far East (6–8). In Israel, TA has been reported in Sephardic but not in Ashkenazi Jews (9). It occurs more frequently in women, who represent up to 90% of cases in adults, while series on childhood TA have shown different female/male ratios: 6.9:1 in Mexico (10), 4.3:1 in Korea (11), 3.2:1 in the USA (12), 2.8:1 in Turkey (13), 1.9:1 in India (14), 1.7:1 in South Africa (15), and 1.2:1 in Israel (16). Although age of onset ranges from infancy to middle age, the highest incidence occurs during the third decade of life (1, 12, 17–22). Some authors have found a double peak-incidence: one at age 10–15 years and a second one at age 20–24 years. Female patients seem to have a major incidence peak between age 15 and 19 years (23, 24). TA is rare but it is the commonest large vessel vasculitis in children, representing the leading cause of stenotic aorto-arteriopathy and one of the most prevalent causes of reno-vascular hypertension in childhood (24, 25). Childhood TA is associated with mortality rates as high as 35% (12, 14, 26, 27).

## Pathogenesis

The etiology of Takayasu arteritis remains poorly understood, but genetic contribution to the disease pathogenesis is supported mainly by its association with the HLA complex. HLA associations are varied and different according to the patients' ethnic background. The strongest association has been established with HLA-B52 in Japanese and other populations (28–30). HLA-B52 positive Japanese patients seem to carry a worse prognosis (31). Additionally, associations with HLA-B5 have been described in patients with Asian and Mexican Mestizo background, HLA-A2, -A9, and -B35 in Arabs and associations with HLA-DR4 have been found in North American patients, although replication studies have shown contradictory results (32–38). Certain polymorphisms (rs12524487 and rs9366782) in HLA-B/MICA have been associated with TA or risk of ischemic brain disease in TA in a Chinese population (39). A study in Indian patients found that the G allele at *TNF-*α-308 was more common in TA patients than in controls, while the A allele was relatively less common in their study subjects than in Western individuals with the disease (40). A large genome-wide association study involving 449 patients with Northern European and Turkish ethnic backgrounds revealed two independent genetic susceptibility loci in the HLA class I and class II regions (*HLA-B/MICA* and *HLA-DQB1/HLA-DRB1)* and a genetic association with other loci, including *FCGR2A/FCGR3A* and *IL12B* (41). A study on Chinese patients found similar associations (42), while Terao et al. reported polymorphisms nearby *IL12B* and *HLA-B* (43). Additionally, a variant in *IL17F* gene (rs763780) has been found to be protective against the development of TA (44).

A pathogenic role for infection has been hypothesized by several investigators, but supporting evidence has so far remained elusive or inconclusive. TA has been reported in HIV patients (45). Watanabe et al. (46) reported on a patient who developed a transient arteritis of both carotid arteries after influenza vaccination. A case of post-hepatitis B vaccination has been described (47). Similarly, the role of tuberculosis (TB) in TA is still controversial. Several published case-series have shown a variable proportion of TA patients who had evidence of preceding or concomitant infection with *Mycobacterium tuberculosis* (17, 27, 48). In a Brazilian study of 71 children with TA, 23 patients (32%) received anti-TB drugs for suspected or diagnosed TB (21). In a short series of Chinese TA patients, 4 out of 9 children had TB before the onset of symptoms (22). A case-control study from Mexico reported the presence of the IS6110 and HupBgene sequences associated with M. tuberculosis within the aortic tissue of TA patients. The authors speculated about the pathogenetic role of TB in the development of arteritis (49). Molecular mimicry between the mycobacterial 65-kDa heat-shock protein (HSP) and human 65-kDa HSP has been suggested, which could elicit an immunologically-mediated cross-reaction and lead to an autoimmune response (50). Several authors have reported the presence of T cells reactive to mycobacterial 65-kDa HSP and its homologous human HSP, as well as serum IgG antibodies directed toward mycobacterial and human 65-kDa HSP, in patients with TA. Furthermore, the 65-kDa HSP has been isolated from the middle layer and vasa vasorum in aortic biopsies from patients with TA (50, 51). Chauhan et al. (52) demonstrated circulating anti-aortic endothelial cell antibodies (AAECAs) that were directed against 60–65 kDa HSP in patients with TA. In this study, sera from AAECA-positive TA patients induced expression of adhesion molecules and secretion of proinflammatory cytokines by aortic endothelial cells, which suggests a potential pathogenic role of these autoantibodies. Finally, the association between TB and TA seems to be much weaker in countries with a low prevalence of TB (53).

**Figure 1:**
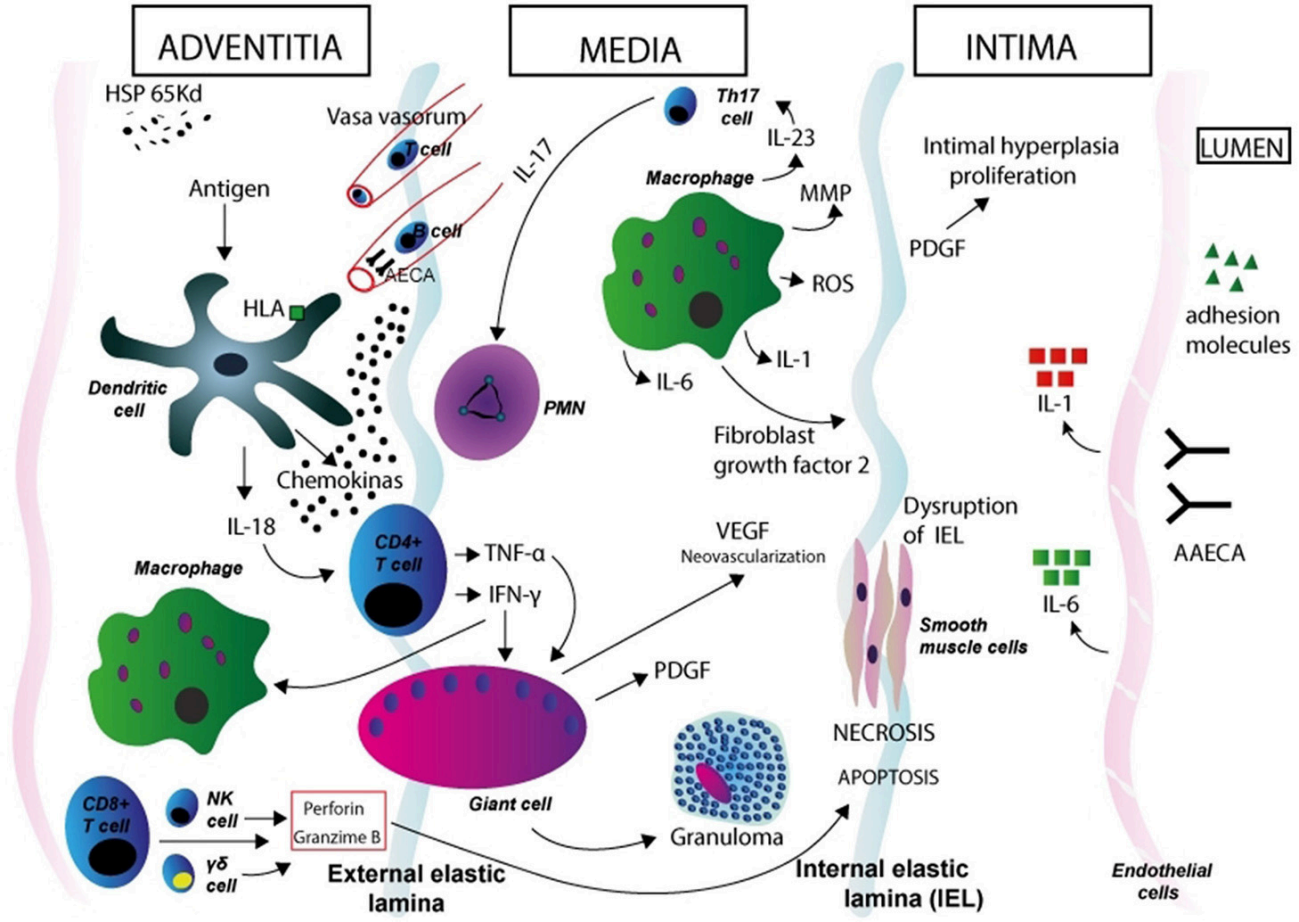
Immunopathogenesis of Takayasu arteritis. Schematic figure showing possible mechanisms in the aortic wall. Dendritic cells in the adventitia expressing specific HLA molecules are activated by a stimulus so far unrecognized. Expression of the 65 kDa HSP in the aortic tissue might play a role in dendritic cell activation. These cells synthesize and release proinflammatory cytokines (such as IL-18) and homing chemokines that recruit T cells to the vessel wall and initiate an aberrant T cell response. After interaction with dendritic cells, CD4-positive T cells with a Th1 phenotype release cytokines such as interferon (IFN)-γ and tumor necrosis factor (TNF)-α, which induce differentiation and increased function of macrophages, and also induce the coalescence of multinucleated giant cells, thus promoting the formation of granuloma. T cells with an induced Th17 phenotype release IL-17, which attracts and activates neutrophils in the vessel wall. Macrophages release IL-1 and IL-6, MMP, and ROS (which induce oxidative injury and degradation of media and intima layers, and disruption of the elastic laminae), VEGF (leading to neoangiogenesis), FGF, and PDGF, which results in exuberant intimal proliferation. These phenomena contribute to the structural damage in the aortic wall. IFN-γ, TNF-α, IL-6, IL-8, IL-17A, and IL-18 likely play a role in vessel wall damage (through the recruitment of mononuclear cells in the vessel wall) and systemic features of TA. CD8-positive T cells, γδ T-cells, and natural killer (NK) cells release of perforin and granzyme-B, which contribute to apoptosis and necrosis of smooth muscle cells and damage in the intimate layer. AAECA may also have a role in pathogenesis through the activation of endothelial cells and induction of complement- and cell-mediated cytotoxicity. Degenerative changes in the media and adventitia, as well as intimal fibrocellular hyperplasia, eventually lead to muscular layer weakening, aneurismal formation, vascular stenosis and thrombus formation. HSP, heat shock protein; HLA, human leukocyte antigen; PMN, polymorphonuclear neutrophil; NK, natural killer cell; MMP, matrix metalloproteinase; ROS, reactive oxygen species; PDGF, platelet-derived growth factor; VEGF, vascular endothelial growth factor; FGF, fibroblast growth factor; AAECA, anti-aortic endothelial cell antibodies.

Different immunological mechanisms are likely involved in TA pathogenesis (Figure 1). Both cell-mediated and humoral immune mechanisms lead to inflammation and tissue damage in TA (54). Both circulating anti-endothelial cell antibodies (AECA) and autoantibody-producing B cell infiltrates in inflamed vessels point to a role of humoral immunity (52, 55, 56). The question of these mechanisms as being pathogenetic or an epiphenomenon remains open. Complement and cell mediated cytotoxicity by AECA have been demonstrated in patients with active disease (57) but these findings have not been replicated so far. Additionally, Hoyer et al. found a significant increase of newly generated plasmablasts in patients with active disease, suggesting a prominent role for B cells in the disease pathogenesis and supporting the use of anti-B cell therapies in TA (58). CD8-positive T cells, the main components of the inflammatory infiltrates in affected vessels, have been proposed as key mediators of vessel damage through the release of perforin and granzyme-B (55). Circulating and tissue-infiltrating γδ T-cells have been reported to be expanded in TA patients during the active phases of the disease (59, 60). It is proposed that dendritic cells, activated by a stimulus so far unrecognized, recruit T cells to the vessel wall. Different cytokines such as interferon (IFN)-γ and tumor necrosis factor (TNF)-α, allow the formation of granuloma. Simultaneously, perforin secreted by cytotoxic Tcells, γδ T-cells, and natural killer (NK) cells, may contribute to the cell damage and necrosis in the medial and intimate layers. (61–63). Proinflammatory cytokines likely play an important role in the pathogenesis (64). Serum levels of IFN-α, TNF-α, interleukin-6 (IL-6), IL-8, IL-17A, and IL-18 are increased in patients with TA (65–69). In particular, serum levels of IL-6, IL-12, and IL-18 correlate with disease activity, while high expression of IL-6 in aortic tissue from TA patients has been reported. (66, 70, 71). Misra et al. showed a significant expansion of Th17 cells and elevated serum IL-17 and IL-23 levels in TA patients as compared to healthy controls (72).

## Clinical manifestations

The clinical manifestations of TA are different according to the time point along the disease course. During the early, actively inflammatory phase, non-specific systemic symptoms predominate for weeks or months, but they are frequently overlooked or considered to be indicative of more common, acute illnesses. During this stage, the disease course may follow a remitting/relapsing fashion, making diagnosis difficult (14, 27). Recurrent disease often occurs in new arterial territories, with the consequent coexistence of active and inactive (sequelae) lesions. The late, chronic phase (the “pulseless” stage) is characterized by ischaemia and symptoms secondary to arterial occlusion.

### General manifestations

Constitutional symptoms are observed in a higher proportion of childhood TA as compared to adult TA. General features are most frequent at onset of the disease and include hypertension in the majority of patients and, in decreasing order of frequency, the following: headaches (31%), fever (29%), dyspnoea (23%), weight loss (22%), vomiting (20%), and musculoskeletal features (myalgia, arthralgia, or arthritis; 14%) (12, 73). However, this non-specific symptoms are rarely disabling.

### Organ-specific manifestations

#### Cardiovascular features

Cardiovascular features have been observed in 75–85% of pediatric patients, hypertension is present in over 80%. Renal artery stenosis may lead to hyper-reninemic, renovascular hypertension, which may be asymptomatic in nearly 50% of patients (25). Systemic hypertension may be associated with a higher risk for arterial vessel wall dissection, a rare but life-threatening complication which is less common in pediatric than in adult TA. Prevalence of dissection in childhood TA has been estimated at 11% (74). Discrepancy of any of four limbs blood pressure >10 mm Hg (which constitutes a classification criteria item) as well as bruits over aorta and its major branches are present in over 50% of patients. Precordial pain, dyspnea, palpitations, and murmurs may reflect cardiac involvement. Dilated cardiomyopathy has been observed concomitantly with hypertension (14). Aortic valve insufficiency and congestive heart failure—an important cause of mortality—have been reported in a significant proportion of patients (73). Additionally, severe chest pain could be the presenting symptom of myocardial infarction—a very rare complication in children, occurring in <5% cases—which can be confirmed on ECGs and/or elevated troponin (75). Pericarditis and valvular heart disease have been reported in a small percentage of patients. Lower-limb claudication due to ischemia is present in 10–35% of childhood TA and may impact daily activities. Carotydinia (pain and tenderness on palpation over carotid bifurcation) is one of the most distinctive symptoms during the acute phases of the disease, but it is uncommon in childhood. Carotidynia may be aggravated by swallowing, coughing, sneezing, or turning the head to the contralateral side (76, 77).

#### Cutaneous features

Cutaneous features have been observed in 2.8–28% patients depending on series and race. Patients with cutaneous features seem to be most frequent in Japan (78). Skin involvement includes: livedo reticularis, purpura, erythema nodosum, subcutaneous edema, urticaria, digital gangrena, and ulcers. In certain cases, ulcerations may resemble pyoderma gangrenosum (79, 80).

#### Neurological features

Nearly 20% of patients evidence neurological involvement, which can be the presenting symptom at disease onset. The most common neurological symptoms are severe headaches; organic confusion, cognitive dysfunction, stroke, meningitis, encephalitis, and seizures (not related to hypertension) may also occur (81, 82). Children with TA exhibit more frequent and heterogeneous neurological features than adults. Intracranial aneurysms have been reported; the middle cerebral artery is the most frequent site of involvement (19, 83, 84). Posterior reversible encephalopathy syndrome, a neuroradiologic condition associated with headache, seizure, visual disturbances, and focal neurological deficit, has been described in childhood TA, albeit rarely (85).

#### Gastrointestinal features

Gastrointestinal involvement occurs in approximately 10% of childhood TA cases. Stenotic arterial segments can cause ischemic symptoms: most frequently, acute or chronic abdominal pain is usually secondary to mesenteric ischemia caused by vasospasm of the damaged intestinal vasculature, which determines reduced blood flow in the intestine during eating (11, 86). Abdominal pain can be associated with vomiting or nausea, blood in the stools, and diarrhea; occasionally, severe pain may reflect bowel perforation. Portal hypertension, an uncommon manifestation, has been reported (87).

#### Ocular features

Visual symptoms can be either transient or persistent and progressive. The ocular manifestations in TA usually follow occlusion or severe stenosis of the carotid arteries, and they commonly appear late during the disease course. Signs of conjunctival and episcleral vascular dilation may occur, but retinal abnormalities are most prominent (88). Complications due to ischemia, vitreous hemorrhage, retinal detachment, or optic atrophy may lead to blindness (88). The most common retinal findings include tortuosity and dilation of retinal veins, arterio-venous shunts, and microaneurysm in the peripheral retina. Acute loss of vision, sometimes associated with orbital pain, has been reported. Vision loss may be secondary to anterior uveitis, cystoid maculopathy, or ischemic optic neuropathy (89).

#### Renal features

Features of renal involvement are rare, and include proteinuria, microscopic hematuria, and decreased glomerular filtration rate secondary to glomerulonephritis. Nephrotic syndrome and IgA nephropathy have been reported (90).

#### Pulmonary features

Pulmonary involvement is uncommon. Pediatric TA patients may exhibit cough and dyspnea; pleural effusion, pulmonary infiltrates, alveolar hemorrhage, respiratory failure, and pulmonary hypertension have been reported in different case series (25, 27).

### Association between childhood TA and other rheumatic/autoinmmune diseases

Different conditions have been reported in patients with TA. Coexistence of inflammatory bowel disease and TA in adult (and rarely in pediatric) patients has been described: almost 10% patients with TA may develop Crohn's disease (CD) or CD-like colitis, indicating an association that exceeds the expected prevalence (91). Although the pathogenesis of both diseases remains unclear some similarities have been found, such as granulomatous vasculitis. Other reported associated diseases are pyoderma gangrenosum, ankylosing spondylitis, and juvenile idiopathic arthritis (92). Vettiyil et al. reported a patient who suffered from all TA, pyoderma gangrenosum, and chronic recurrent multifocal osteomyelitis (93).

## Imaging

**Figure 2:**
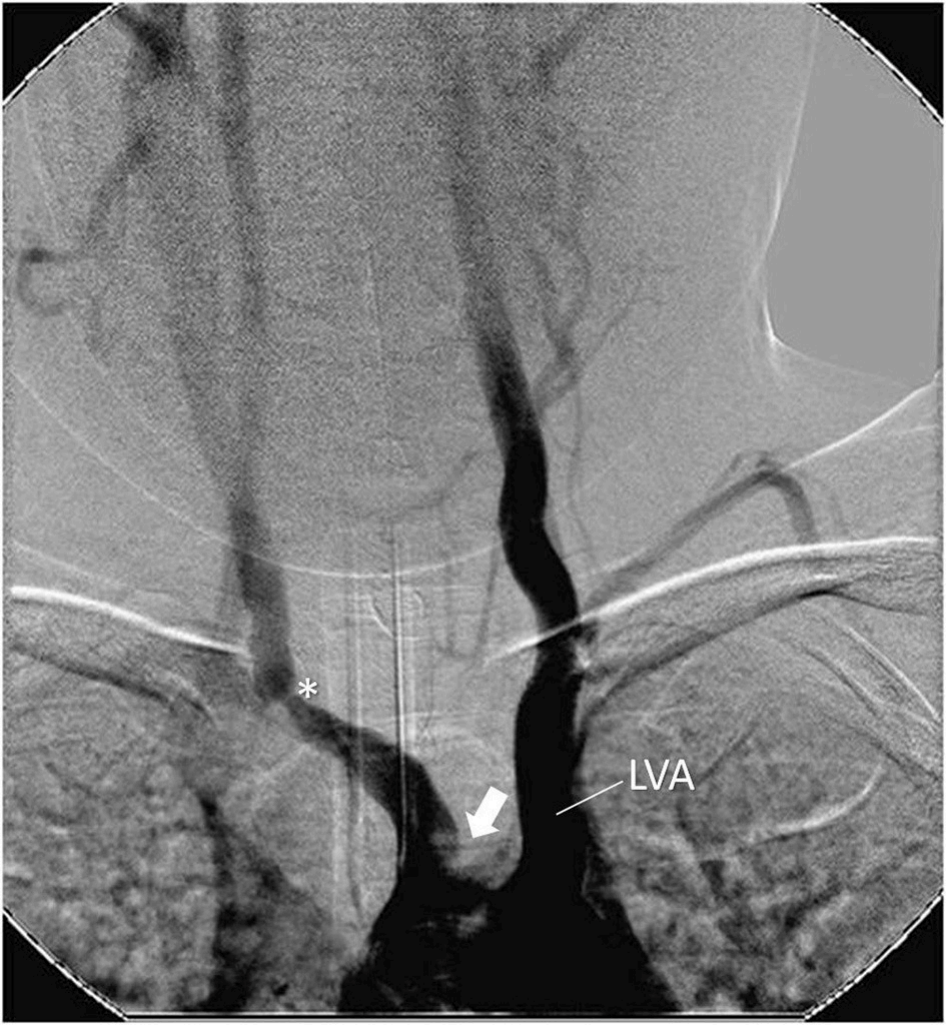
Angiography showing stenosis in the brachiocephalic trunk at the subclavian emergence (*). Occlusion of left carotid artery close to the aortic arch (arrow). Left dilated vertebral artery emerging from the aortic arch (LV). Female, 7 year-old patient with Takayasu arteritis.

**Figure 3:**
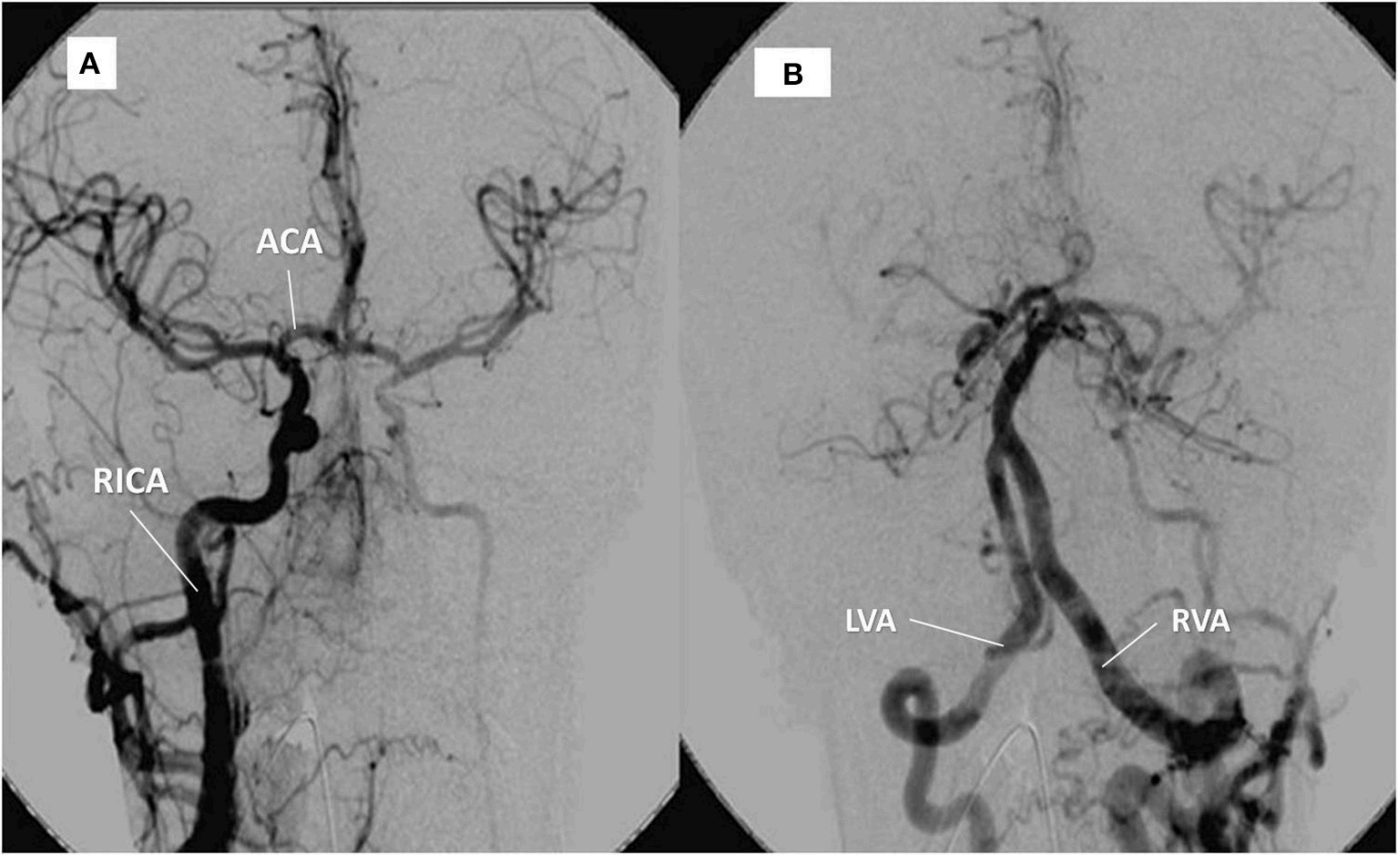
Same patient as in Figure 1. **(A)** right internal carotid artery (RICA) supplying the left hemisphere through the anterior communicating artery (ACA). **(B)** contrast into the left, hypertrophic vertebral artery (LVA) provides supply to the right vertebral artery (RVA) and the territory of the (occluded) left carotid artery.

**Figure 4:**
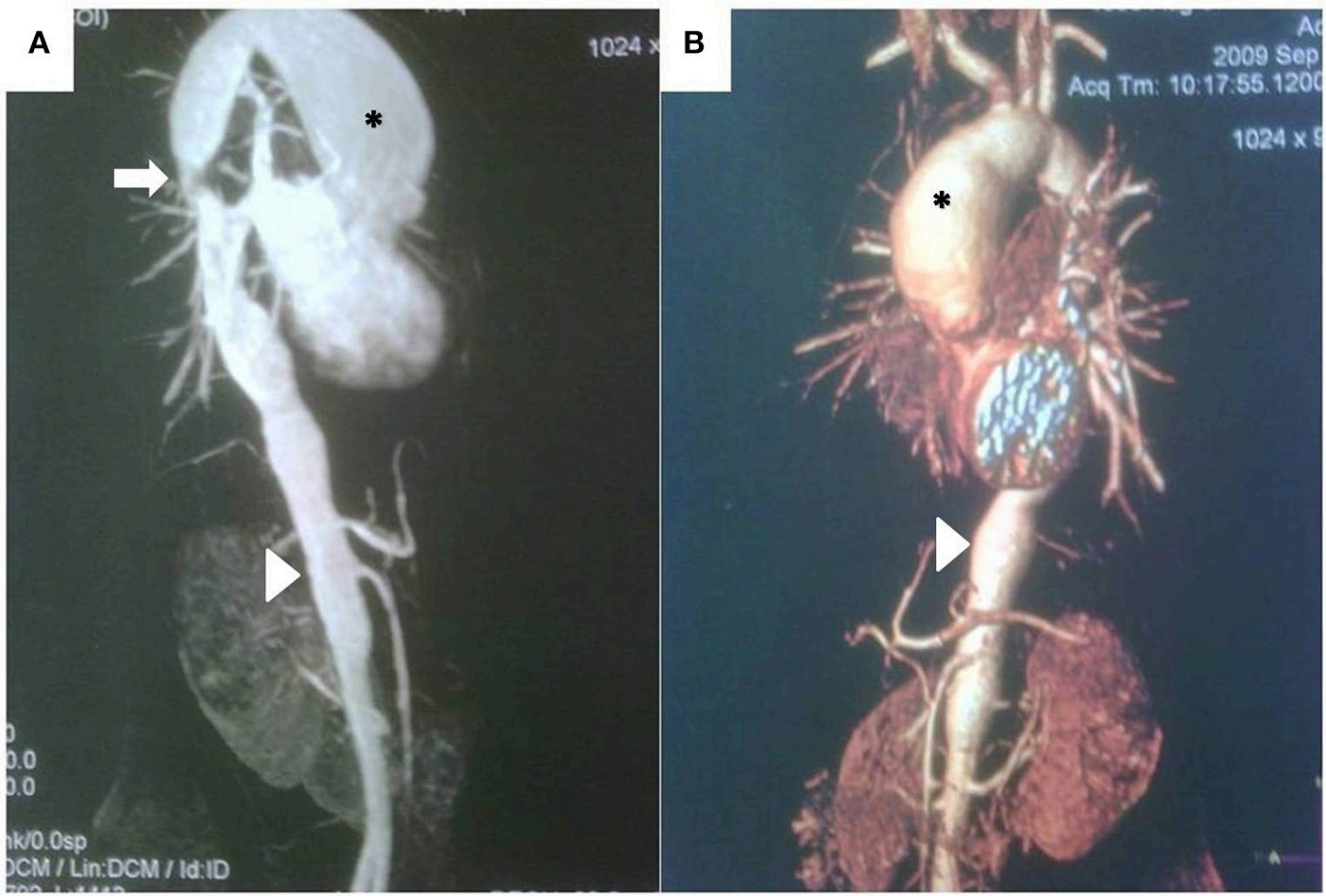
**(A)** Magnetic resonance angiography demonstrating large, secular aneurysm in the aortic arch (*), stenosis in the thoracic aorta (arrow), and irregularity of the thoracic and abdominal aorta, including stenotic areas and a long aneurysm (arrowhead) proximal to the renal arteries in a 13 year-old girl with recent-onset Takayasu arteritis. **(B)** CT scan and three-dimensional reconstruction of the same patient, demonstrating same findings, but providing better quality-anatomical details.

**Figure 5:**
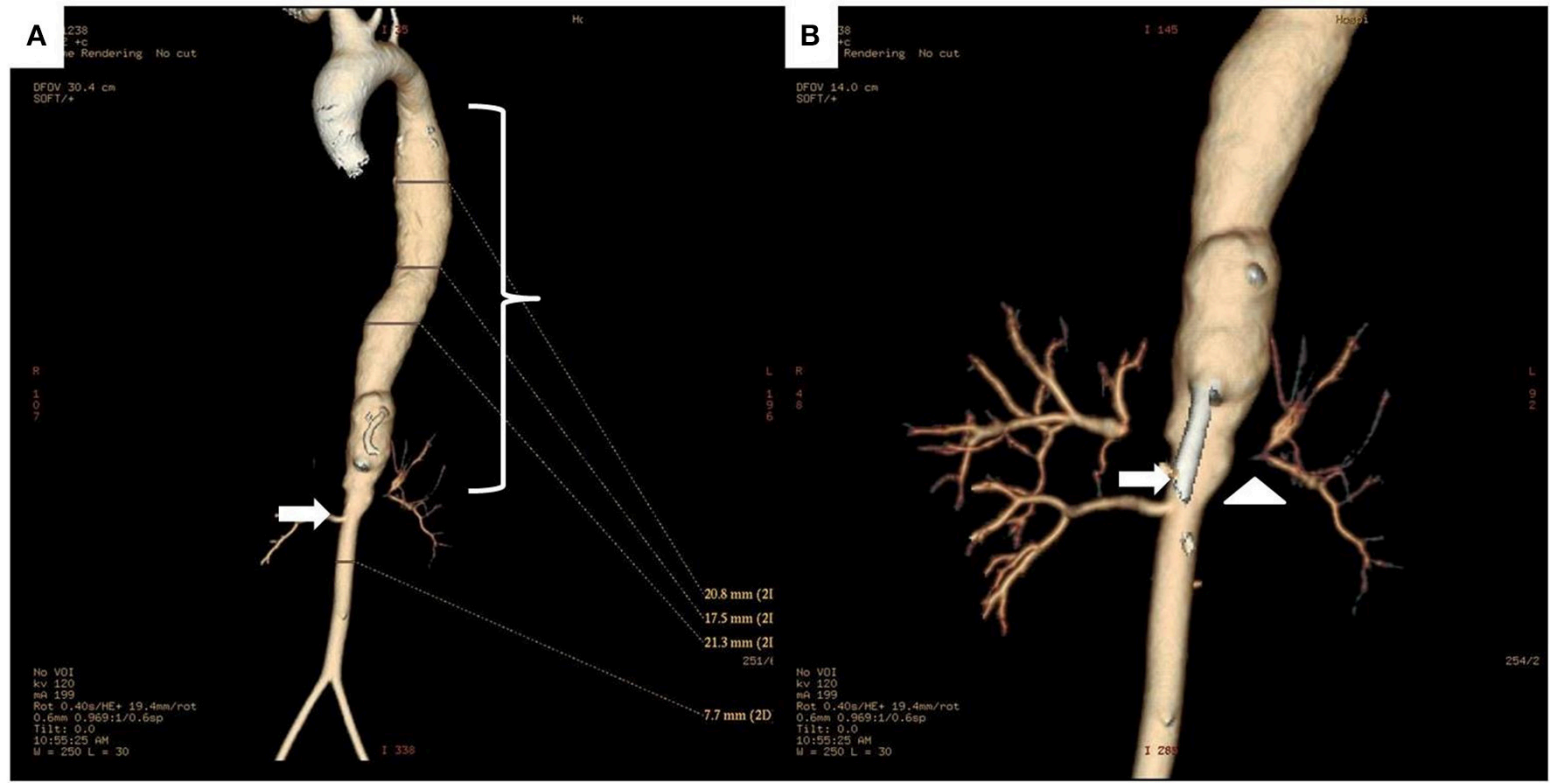
**(A)** 3D reconstruction CT images of the whole aorta in a 12 year-old female Takayasu arteritis patient with thoraco-abdominal aortic aneurysm (bracket) proximal to a stenotic lesion (arrow) at the renal artery emergence site. **(B)** Detail of the aneurysmal dilatation and stenosis of the abdominal aorta (arrow), and stenosis of left renal artery (arrowhead).

Diagnosis and monitoring of TA require imaging of the blood vessels. Conventional angiography, contrast-enhanced magnetic resonance angiography (MRA), and Computed tomography angiography (CTA) offer advantages and disadvantages for the purpose (Table 1). Angiography using digital subtraction technique (DSA), the method used routinely for the evaluation of the arterial tree in patients with suspected or confirmed TA for decades, provides the best assessment of the vessel lumen (not the vessel wall), but it is invasive, it exposes the patient to ionized radiation and it is not devoid of complications risk (Figures 2, 3). For these reasons it is seldom used in clinical practice and non-invasive imaging methods have largely replaced it as a useful tool for diagnosis and follow up of TA patients. MRA has a comparable accuracy and sensitivity to DSA in the assessment of TA, particularly when lesions are in the aorta (Figure 4). It is non-invasive, it does not expose the patient to iodinated contrast load or radiation, and can provide information on arterial wall anatomy (thickness, edema, and contrast enhancement) during active, inflammatory phases on most vessels (94, 95). It is probably the most widely employed method for diagnosing and monitoring disease activity in patients with TA nowadays (it is certainly the technique of choice in the authors' experience) and it has been extensively used in children (96, 97). Diffusion-weighted MRI may be a useful imaging modality to assess the vascular inflammation and discriminate between active and non-active arterial lesions (98). Drawbacks of MRA include its inability to capture small vessels and the possibility of overestimating the degree of vascular stenosis (99). Typical features of childhood TA in angiography and MRA imaging are—similarly to findings in adult patients—stenosis, fusiform dilatations, aortic wall thickening, mural thrombi, and a bright signal in T2-weighted images indicating inflammatory edema of the vessel wall (100, 101). Stenotic lesions are usually found near the origin of the aortic branches; collateral vessels are indicative of the chronicity of stenotic lesions (99, 102, 103). On the other hand, CTA has a better resolution—and it provides excellent anatomic detail in 3D reconstruction images (Figure 5)—, but exposes the patient to high radiation and it is not suit for repeat follow-up assessments. It is certainly helpful when MRA is not available. Both MRA and CTA provide cross-sectional arterial wall images and allow detection of intramural inflammation, and they both demonstrate high specificity and sensitivity for the diagnosis and disease activity assessment (104–106).

**Table 1:** Imaging modalities in the evaluation of Takayasu Arteritis patients.

**Modality**	**Advantages**	**Disadvantages**
Digital substraction angiography	Excellent morphological definition (lumen) Allows vision of distal vessels	Invasive Radiation Low sensitivity for assessment of disease activity Does not provide information about vessel wall
Magnetic resonance angiography	Good morphological definition (lumen) Fairly good measurement of arterial wall thickness Allows vision of arterial wall edema Provides information on gadolinium uptake in vessel wall Good sensitivity for assessment of inflammation No radiation	Does not allows vision of small vessels It may overestimate stenosis Expensive
Computed tomography angiography	Good morphological definition (lumen) Fairly good measurement of arterial wall thickness Good imaging of arterial lumen	Radiation Low sensitivity for assessment of disease activity Does not allow vision of small vessels
Doppler ultrasound	Very good measurement of arterial wall thickness Provides fair definition of anatomical details Inexpensive Fair sensitivity for assessment of disease activity No radiation Non-invasive	Operator-dependant Poor definition of descending aorta No direct measure of inflammation
18F-fluoro-deoxy-glucose positron emission tomography	Good sensitivity for early assessment of inflammation	Very poor definition of anatomic details Radiation Expensive It may underestimate activity

Ultrasound (US) with color Doppler provides information about the morphology of the vessels and can also detect thrombosis and aneurysms, especially in the carotid arteries. It is inexpensive and does not use radiation or contrast, but it is operator-dependent and does not determine disease activity. A diffuse thickening of the intima–media complex, the “Macaroni sign,” has been described in ultrasonographic studies of patients with TA (107). Recent studies have shown that US can provide important information about the vessel wall in pre-stenotic stages, when edema causes the vessel wall to appear hypoechoic, in contrast with its hyperechoic look in basal, non-inflammatory stages (108). US may, therefore, be a valuable method for follow up of TA patients. In the last few years, 18F-fluoro-deoxy-glucose (FDG) positron emission tomography (PET) imaging (18F-FDG-PET) has been added to the set of imaging procedures used in the evaluation of TA. This non-invasive method combines the measurement of metabolic activity of the arterial wall with lumenography, thus providing information about the degree of disease activity and anatomical abnormalities, even before morphologic changes appear on other imaging studies (109). According to some studies, the sensitivity and specificity of this method for clinical activity are close to 100% in TA patients, and it could detect subclinical activity (110–112). Other investigators, however, have found this method to be not as sensitive and specific for disease activity (113, 114). Besides, 18F-FDG-PET is expensive and exposes the patient to high doses of radiation if combined with CT (potentially avoided if MRA is substituted), which limits its use for follow up (115).

The 1994 International TA Conference in Tokyo established an angiographic classification on the basis of the distribution of the lesions (116, 117): type I (branches of the aortic arch, classically associated with the typical pulseless disease), type IIa (ascending aorta, aortic arch and its branches), type IIb (ascending aorta, aortic arch and its branches, and thoracic descending aorta), type III (thoracic descending aorta, abdominal aorta, and/or renal arteries), type IV (abdominal aorta and/or renal arteries), and type V (combined features of types IIb and IV). Gubrandsson et al. proposed an additional category (prestenosis) for patients diagnosed before developing stenosis, based on the finding of abnormalities in the arterial wall or the aorta lumen on MRA, CTA, or PET (53). Ethnic and age-related differences in the pattern of arterial involvement have been reported. Type V and IV seem to be prevalent in children and non-Europeans (12, 21, 22, 27, 53, 118), while Type I may be the most common form in adults and Europeans (119). A large Japanese registry of 1,372 patients showed that the most common angiographic types were I and V, being Type I prevalent in female patients, and type V in male patients. In this registry, patients with younger onset (<40 years) had higher proportions of type I, IIa, and IIb, whereas patients with older onset had a higher proportion of type V and coronary artery lesions (23). A childhood TA series showed type I was the most common form of aortic involvement (13).

In a large Brazilian study involving 71 pediatric patients, imaging on 47 subjects demonstrated at baseline a change in abdominal aorta in 67.2% of patients, renal arteries in 55.2%, and subclavian arteries and descending thoracic aorta in 26.9% each. Stenosis was present in 90%, obstruction in 28.4% and aneurysm in 14.9%. The most frequent angiographic type was type IV (21). In a Canadian study, the most frequently involved vessels were the abdominal aorta (89%), the renal (67%), and carotid arteries (56%). Pulmonary arteries were involved in 19% of cases (48). Left subclavian and common carotid arteries appear to be more frequently involved than their contralateral pairs according to different series from different regions of the world (12, 53, 118, 120).

Finally, the European League Against Rheumatism (EULAR) has recently developed evidence-based recommendations for the use of imaging methods in large vessel vasculitis in clinical practice. MRA should be used as the first imaging test to make a diagnosis of TA and to investigate vessel wall inflammation and/or luminal changes, while PET, CT and/or US may be used as alternative imaging modalities (121). These recommendations also propose MRA, CTA, and/or US as the imaging modalities to be used for long-term monitoring of disease activity and structural damage.

## Pathology

Samples obtained during surgery or autopsy have provided evidence to the pathological findings in arteries of patients with TA (122). All layers of the arterial wall are affected. The initial site of inflammation is the medio-adventitial junction, where vasa vasora penetrate the artery wall. Active lesions usually exhibit edema, inflammatory infiltrates composed of mononuclear cells [dendritic cells, macrophages, NK cells, T cells (αβ, γδ, and cytotoxic T cells)], granulomatous reaction with giant cells, and necrosis in the media and adventitia, as well as intimal fibrocellular hyperplasia and thrombus formation, with ulterior degenerative changes leading to muscular layer weakening and aneurysmal formation. Chronic lesions are characterized by patchy infiltrates containing macrophages, media scarring and fibrosis, which extends to the adventitia. Changes may be diffuse or localized, and obstructive/stenotic lesions are more common than aneurysms in children (123, 124). Also, granulomata may be less common in pediatric patients (27).

## Laboratory and biomarkers

There are no specific laboratory tests for TA or available validated biomarkers of disease activity which could be useful for clinical care or clinical trials. Approximately one third of patients have no elevated inflammatory markers at presentation. Acute phase reactants, such as erythrocyte sedimentation rate (ESR) and C-reactive protein (CRP), are the most valuable non-imaging tests used to monitor disease course, although they correlate with disease activity only in a proportion of patients (1, 3, 125, 126). CRP levels, on the other hand, have been associated with thrombotic events (127, 128). Normocytic, normochromic anemia, leukocytosis, thrombocytosis, and elevated serum amyloid A and fibrinogen may also accompany active phases of the disease. Serum autoantibodies such as AECA, circulating endothelial cells, and serum proteins such as Vascular Endothelial Growth Factor, matrix metalloproteinase-9, IL-6, and IL-18 have been investigated as potential biomarkers for disease activity in TA, but results have been so far inconclusive (66, 129–131). Pentraxin3 (PTX-3) serum levels have been reported to be associated with active disease (132, 133). Also, platelet-to-lymphocyte ratio (PLR) and neutrophil-to-lymphocyte ratio (NLR) have been reported to reflect the inflammatory phases of the disease (134). These findings still require confirmatory studies in clinical settings. Inflammation-induced thrombosis and platelet dysfunction may be common in TA. In a study, patients with TA had higher levels of platelet P-selectin and plasma thromboxane B2, and lower plasma cyclic adenosine monophosphate levels than healthy subjects, which indicated increased platelet activity (135). Akazawa et al. demonstrated that levels of β-thromboglobulin, thrombin/AT-III complex, fibrinopeptide A, and D-dimer are significantly higher in TA patients than in normal controls, reflecting a hypercoagulability state (136).

## Diagnosis and classification criteria

The diagnosis of TA is challenging for the clinician. There are no specific laboratory abnormalities and the disease presentation is often non-specific, silent, or pauci symptomatic. Difficulty in recognizing the disease originates delay in diagnosis—which is close to 2 years median, and commonly several years long in pediatric TA—and consequently a worse prognosis. Approximately one third of children will be diagnosed in the stenotic, pulseless phase of the disease (12, 18, 25–27, 137). Suspicion is usually raised by the presence of hypertension, vascular bruits, asymmetric blood pressure between limbs or asymmetric arterial pulses in extremities, sometimes accompanied by fever, malaise or musculoskeletal symptoms. Even in the active phases of the disease, the laboratory may exhibit increased acute-phase proteins or be completely normal. The diagnosis of TA is based on the demonstration of lesions in the aorta or its major branches. Therefore, evaluation of the entire aorta and its main branches (including cranial vessels, irrespective of the presence of neurological symptoms) should be performed (138). Also, other causes of aortic and large vessel involvement should be excluded (Table 2). The differential diagnoses of TA include congenital disorders (such as aortic coarctation, Marfan syndrome, and fibromuscular dysplasia), other primary vasculitides, and secondary vasculitides [both infectious and autoimmune; (139)].

**Table 2:** Differential diagnosis in Takayasu arteritis.

Systemic infections
HIV Brucellosis Endocarditis
Infectious aortitis
Syphilitic aortitis Tuberculous aortitis
Autoimmune systemic diseases
Primary systemic vasculitis: Kawasaki disease, polyarteritis nodosa Rheumatic fever Systemic Lupus erythematosus Sarcoidosis Spondyloarthropathies Behcet's disease
Non-inflammatory conditions
Ehlers Danlos IV Congenital coarctation of the aorta Fibromuscular dysplasia
Marfan's syndrome

Different sets of diagnostic and classification criteria have been developed in adult patients to differentiate TA from other vasculitides. Their performance in pediatric patients is uncertain. The Ishikawa diagnostic criteria, developed in 96 Japanese TA patients, are based on 3 major and 10 minor criteria, and propose angiography as the main imaging modality to ascertain large vessel involvement (18, 140). The American College of Rheumatology (ACR) classification criteria were also developed to distinguish TA from other vasculitides in adults (141). There are no diagnostic criteria for TA in children. The EULAR/PRINTO/PRES criteria for the classification of childhood TA include angiographic abnormality (conventional, CT, or MRI) of the aorta or its main branches and pulmonary arteries (mandatory criterion) plus at least one of the following: (1) absence of the peripheral artery pulse or claudication induced by physical activity; (2) *a* >10 mm Hg difference in systolic blood pressure in all four limbs; (3) bruits over large arteries; (4) hypertension (when compared with age-matched healthy children); and (5) increased levels of acute phase reactants (ESR and/or CRP). Fulfillment of these criteria provides a sensitivity and specificity over 99% (142).

## Assessing disease activity and damage

Lack of sensitive and specific biomarkers or “gold standard” imaging procedure poses a challenge to clinicians at the time of assessment of disease activity in TA. In practice, periodic examinations using non-invasive imaging methods (namely MRA), coupled with clinical manifestations and acute phase reactants, are commonly sufficient to monitor inflammatory activity and adjust therapy decisions accordingly. However, pathological studies have revealed arterial inflammatory activity in patients whose disease was clinically inactive (1, 13, 143).

Different definitions of “activity,” “remission/relapse,” and scores to quantify disease activity and damage have been proposed by several authors (1, 144–152) (Table 3). One of the most commonly adopted approaches for disease activity assessment in TA is the definition by the US National Institute of Health (NIH) (1): active disease is defined as new onset or worsening of 2 or more from: (a) constitutional symptoms (fever, musculoskeletal pain with no other cause identified), (b) elevated ESR (>20 mm/h), (c) features of vascular ischemia or inflammation (such as claudication, diminished or absent pulse, bruit, vascular pain [carotydinia]), asymmetric blood pressure in either upper or lower limbs [or both]), and (d) new vascular lesions in previously unaffected vessels diagnosed by imaging examinations. Despite its widespread use in clinical settings, this definition has not been validated in pediatric patients yet.

**Table 3:** Disease activity and damage scores used in childhood Takayasu Arteritis.

**Score**	**Activity vs. Damage**	**TA-specific**	**Validated in pediatrics**	**Type of vasculitis**	**Highlights**	**References**
NIH^*^	Activity	Yes	No	Large vessels	^*^It is based on 3 aspects: clinical, laboratory and angiographic ^*^Reproducibility	(1)
BVAS	Activity	No	No	Small and medium vessels	^*^Underestimates cardiovascular findings ^*^Reproducibility	(146)
ITAS2010	Activity	Yes	No	Large vessels	^*^ It is composed of clinical features ^*^It ranks cardiovascular findings ^*^Reproducibility	(150)
ITAS-A	Activity	Yes	No	Large vessels	^*^It is composed of clinical features and acute phase markers ^*^Reproducibility ^*^Sensitive to change	(150)
DEI-TaK	Activity	Yes	No	Large vessels	^*^Only a few features are represented	(149)
PVAS	Activity	No	Yes	Small and medium vessels	^*^Only a few features are represented	(151)
TADS	Damage	Yes	No	Large vessels	^*^It may be optimal for large vessels	(147)
PVDI	Damage	No	Yes	Small and medium vessels	^*^Only a few features are represented ^*^It may not be optimal for large vessels	(152)

* *Definition of activity. TA, Takayasu arteritis; NIH, National Institutes of Health; BVAS, Birmingham Vasculitis Activity Score; ITAS, Indian Takayasu Activity Score; DEI-TaK, Disease-Extent Index for Takayasu arteritis; PVAS, Pediatric Vasculitis Activity Score; TADS, Takayasu Damage Score; PVDI, Pediatric Vasculitis Damage Index*.

The generic Birmingham Vasculitis Activity Score (BVAS) is a validated tool for small-vessel and medium-vessel vasculitis, but it has also been used as an outcome measure in TA by different investigators, both in adult and pediatric cohorts (144–146). The components of BVAS include items indicative of disease activity in organ systems rarely affected in TA, while cardiovascular features (which predominate in TA) are under-represented. Moreover, the absence of imaging data in this tool is a disadvantage in the assessment of disease activity in TA. Overall, the BVAS score is generally considered a tool insufficient for appropriate measurement of activity in TA (147).

A disease-extent index for TA (DEI.Tak), which comprises 59 clinical (not imaging) items based on the BVAS components, was created for the follow-up of patients in 2005 (148). This score, which incorporates symptoms present in the previous 6 months, has been validated in adult TA patients (149). Also, a disease activity index based on items derived from BVAS and weighted for large-vessel vasculitis has been developed. Derived from DEI-Tak, the Indian Takayasu Clinical Activity Score (ITAS2010) (150) measures disease activity through the assessment of symptoms that occurred or worsened over the previous 4 weeks, and are persistent for <3 months. ITAS 2010 includes 44 items, with emphasis on cardiovascular symptoms (33 items). It has good comprehensiveness and the inter-rater agreement is better than a physician's global assessment (PGA). However, convergent validity by comparison to PGA is low. The ITAS-A score incorporates acute phase reactants to the ITAS2010. Both composite, TA-specific disease activity indices (ITAS2010 and ITAS-A) have been validated in adult TA patients, have demonstrated to be sensitive to change, and provide quantitative grading of disease activity (150). These instruments still need age-adaptation and validation in children.

The Pediatric Vasculitis Activity Score (PVAS), which was designed according to the BVAS layout, is the unique validated disease activity tool for children with primary vasculitis (151). However, it may not be optimal for assessment of disease activity in pediatric large vessel vasculitis„ despite patients with pediatric TA having been included in its development process.

Morbidity and mortality are associated with damage secondary to disease activity, therapies or comorbidities in TA. The Takayasu Damage Score (TADS) was derived from DEI.Tak to capture the extent of damage caused by the disease (147). It consists of 42 items in 7 fields, with an emphasis on the cardiovascular system, and records features which have present for at least 3 months. There are limitations in its use due to the difficult differentiation between activity and damage in large vessels. A vascular stenosis may be due to the inflammation taking place in an acute-phase, early state; however, it may also be a sign of an ongoing narrowing of the vessel wall in longstanding disease or the result of scarring. Further research is needed to test such discrimination of outcome tools. On the other hand, the Pediatric Vasculitis Damage Index (PVDI), a generic score developed for children based on the adult Vasculitis Damage Index (VDI), has been used in the assessment of childhood TA. It scores irreversible, cumulative disease-related damage and includes symptoms present for more than 3 months (152).

PVAS, ITAS2010, and PVDI have been used in pediatric TA, although they haven't been validated in this setting yet (22, 27, 48). Specific and useful patient-reported outcomes, assessment of health-related quality of life, and probably additional composite measures for monitoring activity and damage still await development and validation in patients with childhood TA.

Finally, imaging-based scores have been developed. A disease activity score based on Doppler US has showed good correlation with clinical activity as measured by ITAS (153). Recently, a score for assessment of radiologic damage in adult TA patients was developed (154). It is based on the presence and extent of stenosis, occlusion, and aneurysms in large vessels (aorta and it branches and pulmonary arteries) as defined by imaging (MRA or CT).

## Management

Treatment of TA is aimed at controlling vascular inflammation and preventing irreversible organ damage. Early diagnosis and timely, aggressive treatment are important in order to improve chances of a satisfactory outcome. After remission is achieved, treatment needs to be continued in the majority of patients to diminish risk of flares and disease progression. Inactive disease off medication is rare (48) and progression of vessel lesions may occur even during clinically inactive phases of the disease.

### Medical treatment

The EULAR 2009 recommendations for the management of large-vessel vasculitis propose early initiation of corticosteroid therapy for induction of remission, use of immunosuppressive agents as adjunctive therapy, and clinical monitoring of therapy with inflammatory markers as supportive data (155). Treatment recommendations for pediatric patients are lacking, but the results of the SHARE consensus recommendations will be available in the near future (156). General measures include blood pressure control: beta-adrenergic blockers, calcium channel blockers, diuretics or angiotensin converting enzyme (ACE) inhibitors have been used in children (27). ACE inhibitors should be used with caution in patients with renal artery stenosis, because their hyper-reninemic state predisposes them to a sudden drop in blood pressure which may pose risk to organs perfused by stenosed arteries.

There is limited data regarding efficacy of immunosuppressants (IS) in children with TA (Table 4). There have been no randomized therapeutic trials in pediatric patients and most available evidence has been derived from observational studies and from clinical trials performedin adult cohorts (157, 161). Classic IS (and biologic agents in the last decade) have been used in the induction and maintenance therapy of adult and pediatric patients with TA, but except for a few controlled clinical trials, most evidence arises from uncontrolled observations (27). Azathioprine, methotrexate (MTX), mycophenolate mofetil (MMF), leflunomide, and cyclophosphamide (CYC) are the most commonly used drugs used for induction or maintenance of remission in TA (162). During active phases, a combination of high-dose systemic corticosteroids (followed by progressive tapering) and IS is the mainstay of therapy, since corticosteroids alone fail to achieve or maintain remission in the majority of patients (1, 157). Maintenance therapy using IS is frequently necessary. Efficacy outcomes are diverse, and remission occurs in a significant proportion of patients treated with IS as demonstrated by different investigators (157, 163–165). In particular, clinical symptoms are improved while angiographic abnormalities do not regress but halt their progression (166, 167). Ozen et al. successfully and safely treated 6 patients with CYC and systemic corticosteroids for induction and MTX for maintenance of remission, and this has probably been the most widely used regime in pediatric TA so far (158). However, relapses are frequent on traditional IS.

**Table 4:** Efficacy studies in Takayasu arteritis including pediatric patients.

**Agent**	**Dose**	**Treatment duration**	**Patients**	**Design**	**Outcome**	**Reference**
Methotrexate + corticosteroids	10–25 mg/week	34 months*	18 patients (children included)	Open label, prospective	Remission 81%	(157)
CYC followed by MTX + corticosteroids	CYC 1.5–1.7 mg/kg/day, MTX 12.5 mg/m2/week	12 weeks followed by 12–18 months maintenance	6 children	Open label, retrospective	Remission 100%	(158)
Tocilizumab	8 mg/kg every 4 weeks	9.5 months*	4 children	Open label, retrospective	Remission 100%	(159)
Tocilizumab	162 mg SQ	12 months	30 adults, 6 children	RDBPC	Relapse in 44% TCZ, 61% placebo	(160)

CYC, cyclophosphamide; MTX, methotrexate; TCZ, tocilizumab; RDBPC, randomized, double-blind, placebo-controlled. *Median.

The use of biological agents in the treatment of TA has gradually become widespread during the past 15 years. Except for two clinical trials, the evidence comes from case series that have shown the striking benefit of their use in adult and childhood TA. TNF-inhibitors (TNFi; etanercept, and particularly the anti-TNF-α monoclonal antibodies, adalimumab, and infliximab), the IL-6 inhibitor, tocilizumab (TCZ), and B-cell-directed strategies such as the monoclonal anti-CD20 antibody rituximab have been increasingly used in the treatment of TA in children. Anecdotal reports have shown reduction in disease activity or even remission in most pediatric and adult patients who are refractory to other therapies or steroid-resistant/dependent (58, 159, 168–174). Additionally, the flare-free survival time is longer in patients on biologics than in patients on traditional IS (175). In particular, TCZ seems to be effective even when traditional IS and TNFi have failed, according to different case- and small series-reports (70, 159, 172, 176–182). In a small, randomized, controlled trial using subcutaneous TCZ in 36 TA patients (6 of them were children) the primary endpoint (reduction in time to flare) could not be met, but the relapse-free rate showed a trend in favor of treated patients (160). Finally, a recent multicenter, controlled trial involving 34 patients with TA failed to demonstrate the efficacy of abatacept (CTLA4-Ig) in maintaining relapse-free survival over placebo. (183). Additionally, since patients with TA may have a hypercoagulable state, some authors advocate the use of heparin or anti-platelet therapy in order to lower the incidence of ischemic events (136, 184).

### Surgical treatment

Vascular surgery has a relevant adjunctive role in the management of patients with TA; it can reduce mortality and improve the long-term prognosis. According to different series, at least one third of children with TA will require surgical interventions (12, 13, 21, 27, 185). Irreversible stenotic or obstructive vascular lesions with hemodynamic impact require revascularization procedures, which should be performed during the quiescent phases of the disease and only in centers with expertise (155, 186–189). Indications for revascularization also include cerebrovascular disease due to cervicocranial vessel stenosis, coronary artery disease, severe coarctation of the aorta, aortic aneurysms, renovascular hypertension, end-organ ischemia, peripheral limb ischemia, and progressive aneurysm enlargement with risk of rupture or dissection (190).

Percutaneous transluminal angioplasty with balloon or stenting have long been used in TA, but despite providing short term benefit, re-stenosis, and aneurysmal formation are frequent and may occur within 1 or 2 years, and justify new interventions such as bypass surgery (24, 124, 190–192). Percutaneous renal artery stenting has been useful in children with acute renal failure due to severe bilateral renal artery stenosis (193) and it is an effective therapy for the management of refractory renovascular hypertension; aorto-renal bypass and renal unilateral or bilateral autotransplantation are also recommended. (124, 194, 195). Moderate to severe aortic regurgitation, which may lead to cardiac congestive failure, requires early surgical correction, even in very young patients (196).

## Outcome

Recent advances in early diagnosis and effective treatment options have significantly reduced mortality and morbidity in childhood TA, but disease course is still progressive, with repeated flares and the need for continuous IS therapy in over 80% cases (1, 12, 14, 27). Early identification of patients at risk for treatment failure and disease flare is still challenging. Children treated with biologic agents carry significantly better outcomes as compared to children treated with non-biologic therapies. Flare-free survival rates were higher for patients on TNFi or TCZ as compared to non-biologic IS (80 vs. 43% at 2 years, *p* = 0.03) in the case-series by Aeschlimann et al. (48). Moreover, children receiving biologic therapies were more likely to achieve inactive disease at last follow-up than children treated with MTX, azathioprine, or MMF (*p* = 0.02). Damage accrual is frequent and usually reflects vessel stenosis: absent pulses (70%), claudication of the extremities (33%), cerebro-vascular accidents (26%) and seizures (11%) were relatively common during a 2.1 years follow up of childhood TA patients in that series.

Causes of death include complications such as arterial dissection, aortic rupture, uncontrollable hypertension, cardiomyopathy, myocardial infarction, renal failure, and infection (15, 21, 27, 48). Mortality rate ranges between 7 and 35% according to different series (7, 13, 15, 21, 26, 27). Extent of arterial and cardiac involvement, age of the patient, and severity of hypertension have a deep impact on prognosis (13, 25, 26). In pooled adult and childhood TA series, fifteen-year survival rates varied according to the occurrence of arterial complications, valvular heart disease, stroke, heart failure, and renovascular hypertension: they were 66.3% in patients with and 96.4% for those without complications (80, 186, 189, 197).

## Conclusion

Childhood TA is a rare but potentially life-threatening condition. Early diagnosis and timely, appropriate management are of utmost importance to reduce risk of morbidity and damage accrual. Hypertension, fever, unexplained weight loss, fatigue or arthralgia associated with vascular-related findings (such as bruits) may help raise the suspicion and prompt imaging investigations to reach an early diagnosis, which should be made before irreversible changes occur in the affected arteries. Assessment of extent of involvement and disease activity is mandatory for management of all patients. Classical inflammatory markers have limited utility in diagnosis and follow-up, and activity scores have not been validated in pediatric population yet. Non-invasive techniques (such as MRA) should be incorporated into the diagnosis and follow-up workup of patients. The level of evidence for the treatment of childhood TA is low, and it is derived from open studies or case series. Conventional IS combined with corticosteroids are the mainstay of therapy, and in refractory cases, biologic agents (including TNFi and TCZ) should be considered as soon as possible in order to prevent end-organ damage due to ischemia. Revascularization of affected organs using endovascular (stenting or balloon) or bypass interventions are needed when arterial stenosis is severe. Improved awareness, timely diagnosis and incorporation of effective therapies and improved monitoring of the disease activity and response to therapy may result in better outcomes in the future.

## Author contributions

All authors contributed conception and design of the review, wrote sections of the manuscript, contributed to manuscript revision, read and approved the submitted version.

### Conflict of interest statement

The authors declare that the research was conducted in the absence of any commercial or financial relationships that could be construed as a potential conflict of interest.
